# Mind the gap: IgG4-related disease mimicking infectious cerebral mass lesions

**DOI:** 10.1186/s40001-022-00669-0

**Published:** 2022-03-19

**Authors:** Andrea De Maria, Chiara Sepulcri, Stefania Tutino, Federica Briano, Federica Toscanini, Pietro Fiaschi, Gianluigi Zona, Gabriele Gaggero, Matteo Bassetti

**Affiliations:** 1 Clinica Malattie Infettive e Tropicali, DISSAL, University of Genoa, Largo R.Benzi 10, 16124 Genoa, Italy; 2 Dept. of Infectious Diseases, IRCCS Ospedale Policlinico San Martino, Genoa, Italy; 3 Dept. of Neurosurgery, IRCCS Ospedale Policlinico San Martino, Genoa, Italy; 4 DiNOGMI, University of Genova, Genoa, Italy; 5 Dept of Pathology, IRCCS Ospedale Policlinico San Martino, Genoa, Italy

**Keywords:** CNS mass, IgG4-related disease, Abscess, Cerebral, IgG4, MRI

## Abstract

**Background:**

Cerebral intraparenchymal masses represent usually a neoplastic, or infectious differential diagnostic workup in neurology or infectious disease units.

**Case presentation:**

Our patient was an 82-year-old male presenting with seizures, cerebral masses and a history of past treated pulmonary tuberculosis. Initial workup included a differential diagnosis of an infectious mass/multiple abscess. After exclusion of infectious or primary neoplastic origins by negative HIV serology, the absence of immune suppression, endocarditic lesions, negative results of blood cultures and bronchoalveolar lavage, negative cerebrospinal fluid workout on spinal tap led to exclusion of infectious causes. A surgical procedure was performed to access one of the lesions. This yielded a firm, cyst-like mass of histiocytic granulomatous tissue with a conspicuous plasmacellular component and a relevant IgG4 plasmacellular component consistent with IgG4-related disease.

Steroid treatment determined conspicuous improvement and led to discharge of the patient.

**Conclusion:**

Parenchymal IgG4-related disease may be included as a new entity in the differential diagnosis of single or multiple cerebral masses in addition to infectious or neoplastic etiology.

## Background

Central nervous system (CNS) masses often pose a diagnostic challenge to physicians. Differential diagnosis mainly comprises neoplastic lesions and vascular and infectious localizations. Differential diagnosis requires analysis of the imaging features in the context of the clinical presentation of the patient, history, localization and number of lesions [1].

Infections of the CNS presenting as space-occupying lesions gain entry into the CNS through the hematogenous route, seed the parenchyma, and cause tissue destruction with a pathogenetic sequence similar to neoplastic secondary lesions [2]. The widening spectrum of opportunistic and emerging pathogens and sometimes similar imaging and presentation characteristics between neoplastic and infectious mass lesions may at first pose some challenges in their identification that ultimately may require bioptic diagnosis.

Updating the knowledge base of diseases that may present with CNS mass lesions is relevant for differential diagnosis. We report here a patient with a classical presentation at the crossroads of infection and neoplastic CNS mass lesions, which turned out to be the first description of CNS mass localizations of IgG4-related disease, an immune-mediated pathology originally described in Japan [3].

## Case presentation

An 82-year-old male was transferred to our institution from another hospital following the sudden onset of seizures, in absence of fever or other systemic symptoms.

His past medical history comprised pulmonary tuberculosis treated for 9 months with a standard four-drug regimen about 20 years before, chronic obstructive pulmonary disease (COPD), and surgical removal of a localized cutaneous melanoma on the right shoulder 1 year before. He had no history of travel to tropical regions with the exception of a recent stay in southern Italy in possibly endemic *Entamoeba histolytica* areas.

**Fig. 1:**
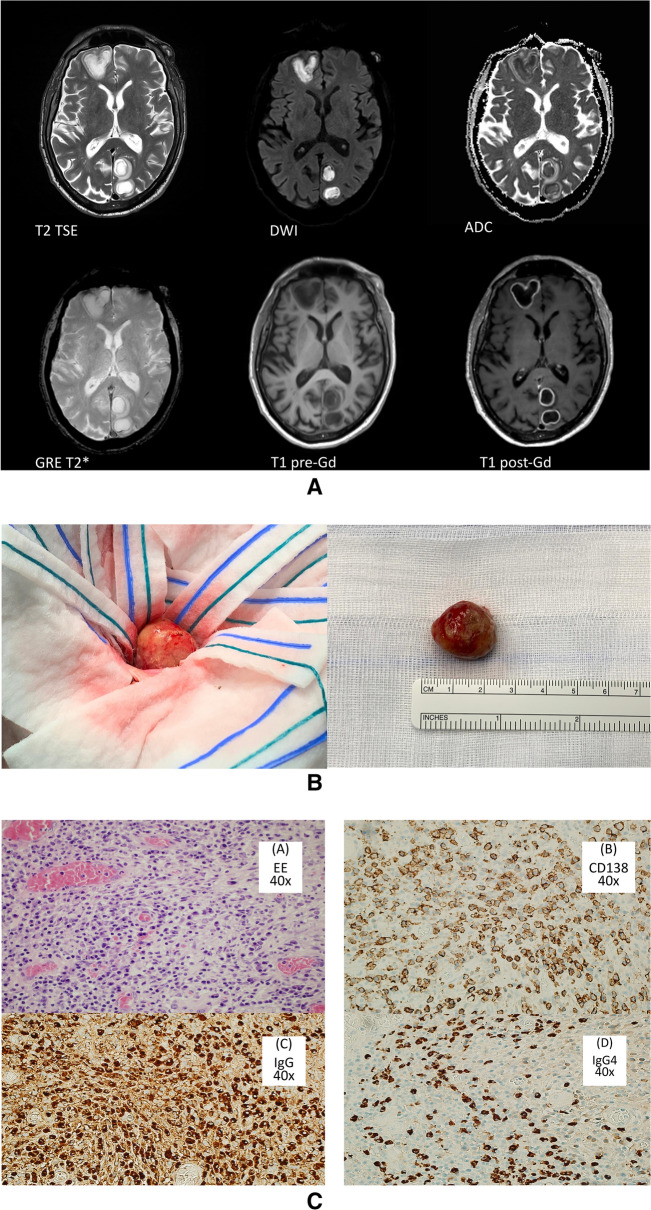
Macroscopic, magnetic resonance imaging and histologic imaging of the CNS space-occupying lesions. Panel **A**: macroscopic appearance of a lesion enucleated during neurosurgical procedure. Panel **B**: MRI shows multiple intraparenchymal subcortical cystic lesions of varying sizes in the right frontal and left temporal lobes. On T2 weighted images these lesions are characterized by a thin rim of intermediate signal intensity and are surrounded by a small area of perifocal high intensity zone most consistent with vasogenic edema. High central intensity on DWI with corresponding low signal on ADC maps reflects restricted diffusion within the lesion. There is a peripheral ring of enhancement on T1 weighted sequences obtained after gadolinium-based contrast agent injection. Panel **C**: histologic analysis of the lesion shown in panel **A**. Figure (**A**): Haematoxylin–Eosin, 40×: (a) a diffuse inflammatory infiltrate composed of histiocytes and less numerous plasma cells, lymphocytes and granulocytes can be seen in a fibrotic tissue, also with incremented vascularization; (b) Immunohistochemistry CD138, 40×: this staining highlights the plasma-cellular infiltrate; (c) Immunohistochemistry IgG, 40×: shows a high number of IgG-positive cells amidst the inflammatory infiltrate; (d) Immunohistochemistry IgG4, 40×: high number of IgG4-positive cells: in a hot-spot of 470 plasma cells more than 10 IgG4 + per HPF can be seen; IgG4/IgG ratio is also increased (45%)

Magnetic resonance imaging (MRI) scan on admission showed multiple CNS lesions in the white matter, with peripheral contrast enhancement, coherent with pyogenic or neoplastic nature (Fig. 1A). Blood tests showed signs of systemic inflammation with leukocytosis, moderate neutrophilia, negative HIV serology, and normal immunoglobulin plasma levels. Echocardiographic findings were negative for vegetations or endocarditis. Serial blood cultures were negative. A positive serology for *E. histolytica* was obtained*,* with negative serologies for *Taenia solium* and *Echinococcus *spp. *Toxoplasma gondii* serology was positive for IgG, negative for IgM. Body temperature and bowel movements were normal. Serum β-d-glucan and galactomannan were undetectable. With a working diagnosis of brain abscess of unknown origin, based on symptoms and MRI, while waiting for blood culture results and trans-esophageal sonographic study, he was started on empiric antibiotic treatment with ceftriaxone and metronidazole and anti-epileptic treatment. Treatment benefit was obtained for the seizures, but not for cerebral mass volume, as shown by sequential CT scans.

A total-body CT scan was performed upon failing improvement of cerebral abscesses, showing bilateral bronchiectasis and a lung cavitation. A diagnostic bronchoscopic procedure was performed with broncho-alveolar lavage. The bronchoalveolar lavage fluid was negative for *Mycobacterium tuberculosis* by PCR and culture, no bacterial or fungal growth was detected, and molecular analysis by PCR was negative for *Pneumocystis jirovecii* and cytomegalovirus.

Tumor markers, autoimmunity tests, and immunological profile (IgA, IgM, IgG count and the flow cytometric lymphocyte subset analysis) were unremarkable. A lumbar puncture was performed. Cerebral spinal fluid (CSF) examination demonstrated a normal cell count, normal glycorrhachia, and an increase in proteins (1103 mg/L) and lactate (2,7 mmol/L). CSF culture for common pathogens as well as PCR for common bacterial and viral pathogens, *T. gondii*, *Cryptococcus* and *E. histolytica* were negative.

To provide diagnostic support for treatment, the right frontal lesion was surgically excised “en bloc.” It presented macroscopically as a firm. elastic, round, well-capsulated mass with a necrotic-like core at post-excision exploration (Fig. 1B). The tissue culture did not provide growth for bacteria, mycobacteria, or fungi. Histology demonstrated the presence of histiocytic granulomatous tissue with a conspicuous plasmacellular component, subsequently defined as an IgG subtype 4 producer (ratio IgG4/IgG 45% on the histological specimen, IgG4 > 10/HPF) (Fig. 1C). Based on these data, a working diagnosis of IgG4-related disease was made, in the absence of other infectious or neoplastic lesions.

The patient was started on steroid treatment (1 mg/Kg of prednisone twice daily) and antibiotic treatment was stopped. Clinical improvement became evident after 10 days of treatment, with this otherwise bedridden patient walking successfully with minimal aid by the second week of treatment.

## Discussion and conclusions

Presentation of the patient at our Infectious Diseases Unit followed standard presentation for a suspected infectious cause of space occupying multiple cerebral parenchymal mass lesions. The initial workout was performed according to standard differential hypothesis and also included initially neoplastic lesions. The final diagnosis was literally surprising, and after a careful literature search resulted to be the first-in-kind.

IgG4-related disease is a systemic fibro-inflammatory disorder originally described by Japanese authors as a form of fibrosing pancreatitis [3, 4] that has been recently individualized [5]. Its organ range has subsequently expanded to encompass heterogeneous extrapancreatic organ involvement, including interstitial pneumonitis, interstitial nephritis, prostatitis, lymphadenopathy, retroperitoneal fibrosis, inflammatory aortic aneurysm, and inflammatory pseudotumor [6]. More recently, cavitating pulmonary disease has been described as mimicking tuberculous disease [7]. Neurologic involvement is infrequent and has been reported mostly outside the blood–brain barrier with intracranial extra-assial masses that eventually represent hypophysitis or pachimeningitis [8–10] mimicking meningioma and never had intraparenchymal appearance.

In the present case, clinical presentation of multiple spherical parenchymal lesions with the initial aspect of abscesses in a man who had been treated for lung TB, a positive serology for *E. histolytica* initially raised difficulties in the diagnostic workup. Histology was helpful to orient the diagnosis and treatment.

To the best of our knowledge, this is the first case of an intra-parenchymal cerebral IgG4-related lesion, while few reports have previously described meningeal or hypophysial involvement [8–10]. Although increased IgG4 levels were not observed in the present case, and there was no apparent pancreatic involvement, several aspects support the diagnosis of CNS IgG4-related disease, including the histopathological result of the lesion’s biopsy, the exclusion of other infectious or neoplastic causes, and the observation of non-TB associated lung cavitation independent of any other infection in line with such reports in IgG4-related disease [7]. In addition, clinical improvement following steroid treatment, as in the present disease course, has been considered as an optional criterion by some authors in IgG4-related disease diagnosis [11]. Indeed, steroid treatment, which is mostly avoided for infectious causes of cerebral abscesses, is currently the initial treatment for this disease, although relapses occur and new therapeutic strategies, including the use of rituximab, have been proposed [12].

In view of the present observation, we suggest that IgG4-related disease may constitute a confounding pathology in the diagnostic workup of presumed cerebral abscess/mass, and may need to be remembered/included in the differential diagnosis of these conditions. The first description in its kind like the present may simply reflect previous underreporting or underdiagnosis, and could represent a gap to mind for infectious disease units caring for these patients.

## Data Availability

Not applicable.

## References

[1] Smirniotopoulos JG, Jäger HR, Hodler J, Kubik-Huch RA, von Schulthess GK (2020). IDKD springer series differential diagnosis of intracranial masses. Diseases of the brain, head and neck, spine 2020–2023: diagnostic imaging.

[2] Santosh V, Mahadevan A, Chickabasaviah YT, Bharath RD, Krishna SS (2010). Infectious lesions mimicking central nervous system neoplasms. Semin Diagn Pathol.

[3] Masaki Y, Kurose N, Umehara H (2011). IgG4-related disease: a novel lymphoproliferative disorder discovered and established in Japan in the 21st century. J Clin Exp Hematop.

[4] Hamano H, Kawa S, Horiuchi A, Unno H, Furuya N, Akamatsu T (2001). High serum IgG4 concentrations in patients with sclerosing pancreatitis. N Engl J Med.

[5] Deshpande V, Zen Y, Chan JKC, Yi EE, Sato Y, Yoshino T (2012). Consensus statement on the pathology of IgG4-related disease. Mod Pathol.

[6] Umehara H, Okazaki K, Masaki Y, Kawano M, Yamamoto M, Saeki T (2012). A novel clinical entity, IgG4-related disease (IgG4RD): general concept and details. Mod Rheumatol.

[7] Jinnur PK, Yi ES, Ryu JH, Iyer VN (2015). Cavitating lung disease: a novel presentation of IgG4-related disease. Am J Case Rep.

[8] Chan SK, Cheuk W, Chan KT, Chan JK (2009). IgG4-related sclerosing pachymeningitis: a previously unrecognized form of central nervous system involvement in IgG4-related sclerosing disease. Am J Surg Pathol.

[9] Shimatsu A, Oki Y, Fujisawa I, Sano T (2009). Pituitary and stalk lesions (infundibulo-hypophysitis) associated with immunoglobulin G4-related systemic disease: an emerging clinical entity. Endocr J.

[10] Goulam-Houssein S, Grenville JL, Mastrocostas K, Munoz DG, Lin A, Bharatha A (2018). IgG4-related intracranial disease. Neuroradiol J.

[11] Shimosegawa T, Chari ST, Frulloni L, Kamisawa T, Kawa S, Mino-Kenudson M (2011). International consensus diagnostic criteria for autoimmune pancreatitis: guidelines of the International Association of Pancreatology. Pancreas.

[12] Ebbo M, Grados A, Samson M, Groh M, Loundou A, Rigolet A (2017). Long-term efficacy and safety of rituximab in IgG4-related disease: data from a French nationwide study of thirty-three patients. PLoS ONE.

